# Obituary

**Published:** 1910-02-26

**Authors:** 


### OBITUARY.

The death is announced of Surgeon-General Alfred Eteson, C.B., at the age of seventy-seven. He took the degree of M.D. at St. Andrews University in 1878, twenty-four years after becoming a member of the R.C.S. of England.

The death is reported of Mr. Ernest Herbert Cobb at the age of forty-nine. Mr. Cobb, a student of St. Thomas's, took his diploma in 1897, was house surgeon at one time and pathological assistant at St. Thomas's. He had been also house surgeon at the Victoria Hospital, Folkestone.

We have to record the death of Mr. Thomas Kilner Clarke, M.A., J.P., aged sixty-six. Dr. Clarke studied at Cambridge, Paris, and Guy's, and was an M.A. and M.B. of the former University. In 1871 he took his M.R.C.S. and became a Fellow the next year. Besides being ex-president of many medical societies he was surgeon to the Huddersfield Infirmary, and at one time was house physician to the London Fever Hospital, and clinical assistant to the Evelina Hospital for Children. He was also a contributor to British and one French medical newspaper.

The death of Mr. H. Dudley Ryder is regrettable from the knowledge that it might have been prevented had he adopted the advice of his medical attendant and submitted himself to operation when the first symptoms of appendicitis manifested themselves. Mr. Dudley Ryder was at one time a student of medicine at King's College Hospital, and filled the positions of house surgeon and resident assistant accoucheur at that institution. He abandoned the medical profession owing to protracted illness. Subsequently he became assistant secretary to the City Carlton Club, and in 1897 was appointed to succeed the late Captain Storrar Smith as secretary to the City of London Hospital for Diseases of the Chest, Victoria Park. At the time of Mr. Ryder's appointment the finances of this hospital were at a low ebb. For some years the revenue had continuously fallen, until the total charitable income in 1897 was only £6,684. The hospital contained 164 beds, but the average number occupied had been reduced to 80. Mr. Dudley Ryder set to work with great energy, and by 1903 the charitable revenue of the hospital had increased to £10,051, and the number of occupied beds to 126. Such a result in a decade which has not been easy for charitable purposes speaks well for Mr. Ryder's ability and value as a hospital secretary. It will be noticed that there are still thirty-eight vacant beds to be filled, a fact which, coupled with the importance and value of the City of London Chest Hospital, should lead to keen competition for the vacant post created by the death of Mr. Dudley Ryder.

